# Comparison of Graph Fitting and Sparse Deep Learning Model for Robot Pose Estimation

**DOI:** 10.3390/s22176518

**Published:** 2022-08-29

**Authors:** Jan Rodziewicz-Bielewicz, Marcin Korzeń

**Affiliations:** Faculty of Computer Science and Information Technology, West Pomeranian University of Technology in Szczecin, ul. Żołnierska 49, 71-210 Szczecin, Poland

**Keywords:** robot tracking, arm tracking, pose fitting, pose estimation, sparse deep learning, sparse CNN, computer vision, depth camera

## Abstract

The paper presents a simple, yet robust computer vision system for robot arm tracking with the use of RGB-D cameras. Tracking means to measure in real time the robot state given by three angles and with known restrictions about the robot geometry. The tracking system consists of two parts: image preprocessing and machine learning. In the machine learning part, we compare two approaches: fitting the robot pose to the point cloud and fitting the convolutional neural network model to the sparse 3D depth images. The advantage of the presented approach is direct use of the point cloud transformed to the sparse image in the network input and use of sparse convolutional and pooling layers (sparse CNN). The experiments confirm that the robot tracking is performed in real time and with an accuracy comparable to the accuracy of the depth sensor.

## 1. Introduction

**Figure 1:**
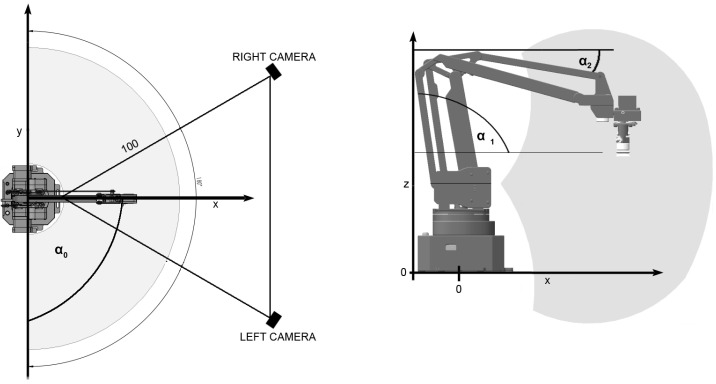
Robot geometry, coordinates: x,y,z, angles: $\alpha_{0},\alpha_{1},\alpha_{2}$; the placement of cameras during the experiment.

The main object under study is uArm Swift Pro [1], the robotic arm with three degrees of freedom (3DOF). The three angles describe the arm state: $\alpha_{0},\alpha_{1}$ and $\alpha_{2}$, as shown in Figure 1. We assume the known restrictions about the robot geometry. The robotic arm consists of two links with knowledge about the dimensions of arms. The main part of the observer is Intel RealSense depth camera D-435 [2]. It consists of two imagers: a depth sensor (enabling depth vision with a range of up to 10 m) and an RGB camera. The research aims to obtain the robot state based on RGB-D images as fast and accurately as possible. The main application of this research may be an additional tool for tracking the robot behaviour in the workspace, e.g., for safety purposes [3]. The second application is to provide a general-purpose tool for tracking skeleton-type objects. In the second approach, we obtain a convenient tool for testing the accuracy of visual tracking because the robot API provides feedback information about the robot arm state. The tracking system consists of image preprocessing and machine learning parts. In the machine learning part, we compare two approaches: fitting the robot pose and fitting a convolutional neural network (CNN). Input images for machine learning algorithms are transformed into the 3D point cloud and into sparse 3D depth images in the next step. In such a case, the use of sparse convolutional and pooling layers is natural and convenient, and it also can help significantly reduce the time of computations.

### Related Research

The task of tracking the robot arm is far from new. Many tracking systems use depth sensors and RGB-D cameras [3,4,5,6]. The main challenges in pose estimation concern the time and accuracy of estimation. Similar techniques are used for human body tracking [7,8]. For example, in [9], a body tracking system with accuracy at level 4–10 cm is reported, but the reported computation time is about 60 s per frame. In [10], human body tracking with multiple Kinect sensors is presented. The reported accuracy is about 10cm (per node) and with a speed of about 25 FPS (frames per s). In [11], the skeleton-based hand tracking system with a processing time of 15 FPS is reported (see also [12]). Hand tracking and gesture recognition may use depth sensors and RGB cameras [13,14,15]. A skeleton-based approach and graph fitting is one of the oldest approaches to body tracking and were used by many authors, e.g., [16]. Their main weakness is the time of computation for more complicated structures. A classification or regression-based approach may work faster, but the learning part is a supervised process that needs properly labelled data.

The presented approach uses RGB-D cameras with an additional depth channel. Another possible approach to tracking objects is applying an event camera (or neuromorphic camera). Compared to RGB-D a, such cameras are not so popular, and they are also difficult to obtain and more expensive. With additional preprocessing steps, such cameras can also provide depth estimation [17].

A neuromorphic camera characterises high dynamic range and low power and bandwidth and power as the signal encoding is intrinsically compressed at the acquisition level. The sensor also achieves a very high frame rate. The output of the event camera only registers independent pixels that respond asynchronously to relative contrast changes, not the full array of pixels. Event cameras are also effectively used for robot tracking purposes, enabling tracking with a theoretically higher speed [18,19].

In the case of the used D-435 sensor, the advantages are a low cost, HD resolution and a direct depth channel. The disadvantage is a low frame rate, which is a limitation in the presented approach.

Another restriction of the presented approach is using an exterior camera that sees the entire work area. There are also approaches using an end-effector mounted camera. Such cameras can also be used to recover the robot position [20]. However, generally, such end-effector mounted cameras are used for other tasks such as object recognition or 3D scanning [21].

Sparse signals are a natural representation of images obtained from depth sensors. Using sparse layers and inputs in CNNs is not as popular as dense ones, but it has also been considered (e.g., [22,23]). Some kinds of sparsity are available in commonly used deep learning libraries. For example, PyTorch [24] provides some functionalities for sparse matrices computations. Tensorflow [25] also provides simple operations for sparse signals, but without the implementation of sparse convolutional layers. A functionality of PyTorch for sparse layers may be extended by using external libraries, e.g., spconv [26] or SparseConvNet [27]. Recent approaches using convolutional neural networks take a point cloud directly on the input [28,29,30,31,32,33]. Such structures may be used effectively for both segmentation and recognition purposes. Contrary to point-based methods such as [31], we use a rather volumetric-based method as proposed in [32]. The main weakness of volumetric-based methods is their computational complexity when using a dense representation.

In our approach, we also put the point clouds into the 3D sparse matrix and use it as sparse input to a sparse layer of a CNN. A CNN-based regression model predicts the state of the robotic arm. The presented solution shows that input images in the form of a point cloud can be handled effectively using existing solutions and tools with sparse layers.

In the following section, we present the experiment’s theoretical background and settings.

## 2. Materials and Methods

**Figure 2:**
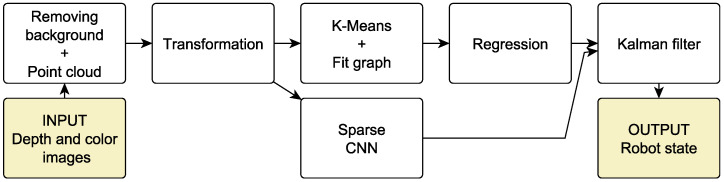
The sequence of the image preprocessing and tracking steps for graph fitting and a convolutional network.

The following section describes the preprocessing details and two considered approaches: fitting the robot pose to the point cloud and fitting the sparse CNN. The whole sequence of the image preprocessing and tracking steps is presented in Figure 2.

### 2.1. Image Preprocessing

Image preprocessing is a common procedure for either graph fitting or deep learning. Preprocessing an RGB image consists of the following steps: (1) removing the background from an image, (2) transforming to a point cloud and (3) unifying coordinates for independence from the camera perspective.

An initial part of preprocessing is removing the background. We use our own clustering-based approach (e.g., [34,35,36]). In our experiments, it worked better than the online learned background subtractor from OpenCV [37]. Using the *k*-means algorithm is simple and efficient algorithm for clustering [38]. The algorithm minimises the inertia criterion:

(1) $$\begin{array}{c} \sum_{x\in X}\min_{\mu \in C}\left({∥x-\mu ∥}^{2}\right), \end{array}$$

where *C* is a set of clusters (each cluster is represented by mean μ). The minimisation is performed in two repeating steps: (1) Assign each sample *x* to the closest cluster centre; (2) update the cluster parameters by averaging the coordinates of all of the samples assigned to the cluster.

In the beginning, we learn the subtractor uses *k*-means with two clusters (k=2) and we identify clusters connected with the background (negative centres) and clusters associated with motion (positive centres). Background and motion masks are fitted on grey-scaled images, and the result is applied to the depth image.

**Figure 3:**
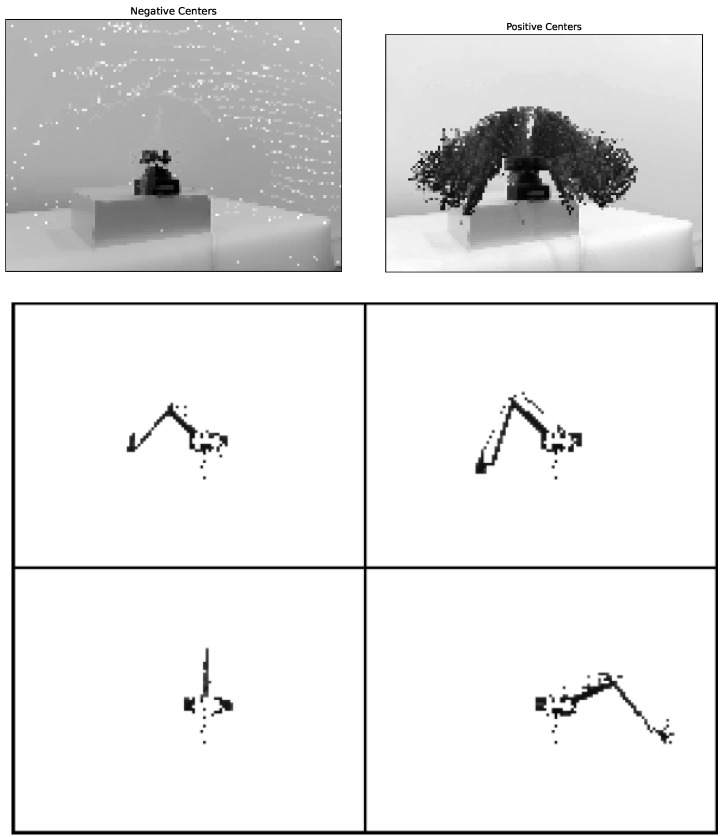
Learning background subtractor. The images at the top are positive (motion) and negative (background) centres and below are exemplary frames after background removal.

In the next step, we remove the background using a static mask for positive and negative clusters. We treat such pixels as the background when a distance between negative and positive centres is less than the threshold. The selected threshold depends on a within-cluster variance. When the pixel distance from the background mask is greater than the threshold, such pixel is treated as in motion. The whole procedure is presented in Table 1. The procedure’s illustration and the exemplary result are shown in Figure 3.

**Figure 4:**
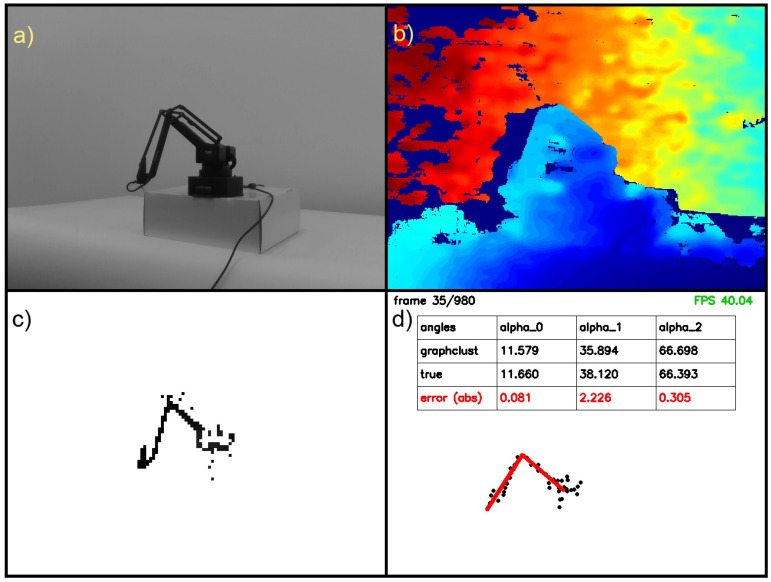
Preprocessing steps: (**a**) input RGB image, (**b**) input depth image, (**c**) image after removing the static background and (**d**) results after transformation to point cloud, *k*-means (black dots) and the result of graph fitting (red lines).

**Figure 5:**
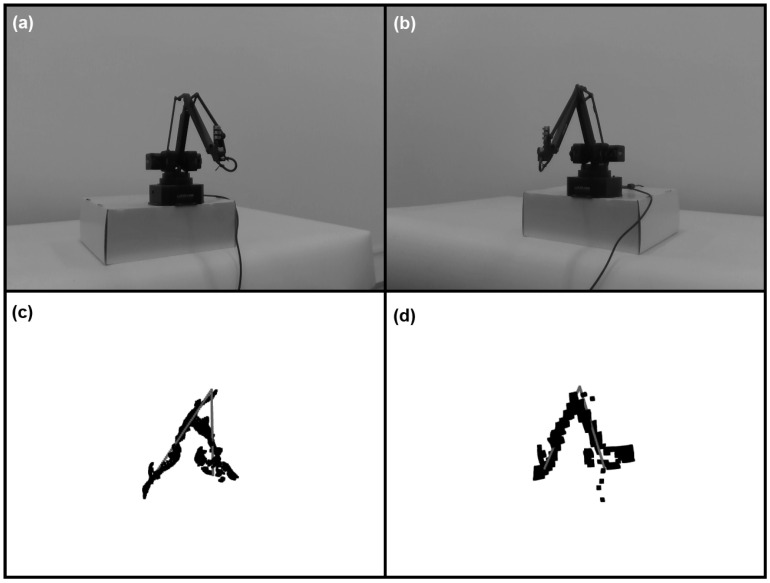
Transformation to the coordinate system independent on the camera. View of the same frame: (**a**) left camera view, (**b**) right camera view, (**c**) point cloud obtained from the left camera after projection and (**d**) Point cloud obtained from the right camera after projection.

After removing a static background, the final step of the image preprocessing is a transformation of the depth image to the points cloud. The 3D point cloud is the input for graph fitting and sparse CNN. We perform a simple calibration and the projection to a unified coordinate system independent of the camera to unify views from two cameras. We perform the calibration process in the following way. The robot arm stays in a given position (e.g., $\alpha_{0}=0^{\circ },\alpha_{1}=90^{\circ },\alpha_{2}=0^{\circ }$), then we fit the graph to the point cloud of the arm. Next, based on points graph coordinates, we produce an orthogonal transformation matrix to the coordinate system independent of the view from a camera. The last step of the preprocessing procedure is projecting the 3D point cloud to the unified coordinate system. We present in Figure 4 images obtained on selected preprocessing steps. An exemplary view after projection to the camera independent coordinate system is shown in Figure 5. After removing the static background and transforming a depth image to the point cloud, we obtain about 3000–5000 points per frame.

### 2.2. Graph Fitting

The graph fitting approach, presented here, consists of two steps. Unsupervised, the first one fits the graph pose to the point cloud. The second one is the supervised correction of estimated angles using feedback information about robot states.

Let G=(E,C) be a graph with vertices (nodes) *C* and edges *E*. The graph fitting procedure aims to fit graph nodes *C* to the point cloud *X* and with a given set of edges *E*. The graph fitting task does not require such a large number of input points. The number of points in the point cloud can be reduced in different ways. The simplest methods are resampling or using the *k*-means algorithm. In the presented approach, we use the *k*–means algorithm—this is a time–acceptable approach, giving more accurate results. The influence of the number of points used in the *k*–means algorithm on the accuracy and the time of computations are presented in Table 2.

The points remaining after the *k*-means procedure can be used to find a graph representation of the robot pose. The aforementioned graph G=(E,C) spans the set of nodes $C=c_{1},\ldots ,c_{k}$ with edges *E*. The edges define robot arm structure. We assume that set of edges *E*—i.e., the structure of the robot skeleton—is known. For a given point cloud $X=x_{1},x_{2},\ldots ,x_{n}$ by *graph fitting*, we mean a geometrical fitting of the graph structure (spanned on nodes *C*) to given points *X*.

Many different measures for the closeness of the graph skeleton to the point cloud during the optimisation procedure were considered. In this paper, we do it as follows: in the first step, we extend the set of graph nodes *C* by adding linearly spaced points on the graph’s edges. This operation gives an extended set of points $C_{ext}$. Next, we use quantity as the closeness measure:

(2) $$\begin{array}{c} dist\left(C_{ext},X\right)=\left\{\;\sum_{x\in X}\min_{c\in C_{ext}}\;d_{2}\left(c,x\right)+\sum_{c\in C_{ext}}\min_{x\in X}\;d_{2}\left(c,x\right)\;\right\}. \end{array}$$

**Figure 6:**
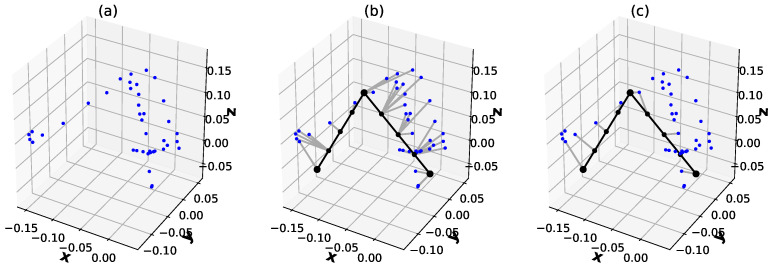
The quality measure for the graph fitting procedure. Robot arm pose (black), graph nodes (black dots), reference point cloud ((**a**), blue dots), extended graph points $C_{ext}$ (smaller black dots). The closeness of the robot pose to the point cloud is the sum of minimal distances between graph nodes to the point cloud (gray lines, (**b**)) and minimal distances between the point cloud to the graph nodes (blue lines, (**c**)).

This quantity is a bit similar to the Hausdorff metric with ∑ instead of max. A computation illustration of dist(C,X) is shown in Figure 6. Using such a quality function, we may use here any optimisation procedure.

We can use output points from the graph fitting procedure (graph nodes *C*) directly to determine the robot arm state (given by angles $\alpha_{0},\alpha_{1},\alpha_{2}$) via geometric constraints. However, it is a bit inaccurate, mainly since the depth sensor provides only the view from one side of the arm. We fit the nonlinear regressor based on points obtained from the graph fitting procedure and take the given robot states as output to correct the robot state estimations.

**Figure 7:**
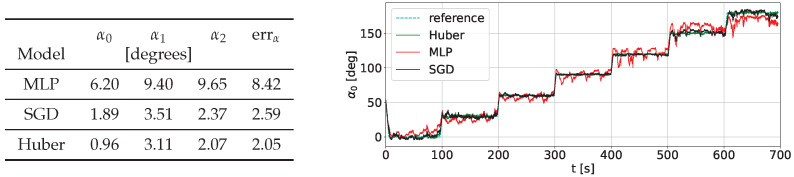
A comparison of accuracies for considered regressors with a different kernel transformation (thanks to [40]): MLP (tanh kernel with linear output layer, using MSE criterion), SGD (RBF kernels with the Nyström method with SGDRegressor using MSE criterion), Huber (RBF kernels with the Nyström method kernel with robust Huber linear regressor).

We considered the usage of two variants of regressors included in the scikit-learn package: two-layer perceptron (learned with MSE criterion) and Huber regressor (learned with approximated MAE criterion) with a nonlinear part. Our regressor experiments show that the Huber regressor is more robust to outliers. As a nonlinear part, we used RBF kernels with the Nyström method [39]. A detailed comparison of accuracy for different kernel regressors is presented in Figure 7. As one can see, the Huber regression with nonlinear kernels attains the best results.

### 2.3. Sparse Convolutional Neural Network

The second model is a CNN-based regressor that predicts the robot state using the point cloud obtained from the preprocessing part. The point cloud is transformed into a 3D sparse array using voxel-based representation. We divide the point cloud space into 4×4×4 mm cubes and set the nonzero value of the matrix element if the responding cube contains a point from the cloud. As a result, we obtain a sparse array with the dimensions 750×750×1375, which is the input for the network.

**Figure 8:**
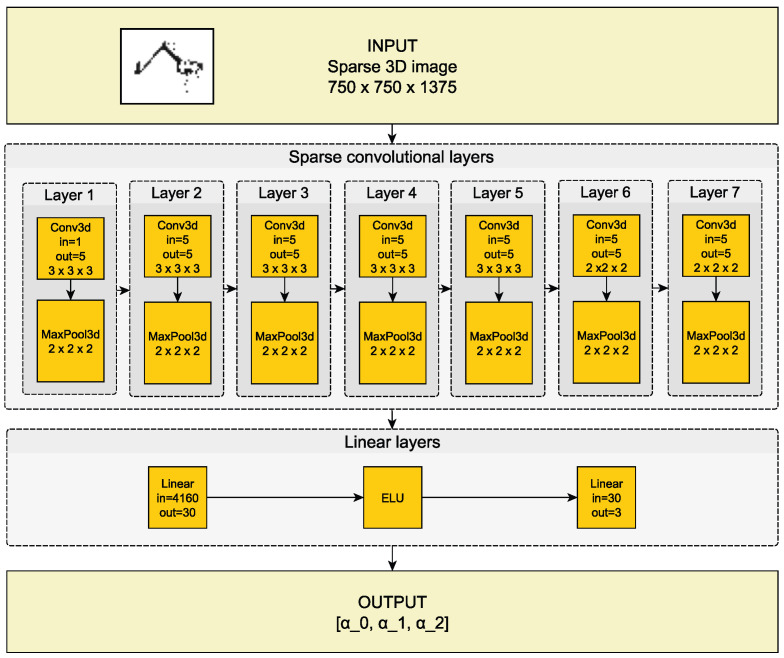
Structure of a sparse convolutional neural network with a sparse image in input and the robot state (angles $\alpha_{0}$, $\alpha_{1}$, $\alpha_{2}$) on the output.

Figure 8 presents the complete structure of the sparse convolutional neural network. The output is predictions of angles that determine the robot’s state. Knowing the robot geometry and using the forward kinematics, we can quickly obtain the position of interesting nodes.

The last tracking step is smoothing measurements using the Kalman filter [41], a standard procedure for smoothing signals produced by a linear dynamical system disturbed by the normal noise. The procedure assumes the system in the form:

(3) $$\begin{array}{cc} x_{t+1} & =A_{t}x_{t}+w_{t} \end{array}$$

(4) $$\begin{array}{cc} z_{t} & =C_{t}x_{t}+v_{t} \end{array}$$

where *A* is the state transition matrix, *C* is the observation matrix and $w_{t}∼\mathrm{Normal}\left(0,Q_{t}\right)$, $v_{t}∼\mathrm{Normal}\left(0,R_{t}\right)$ are random state and observation noises. The filter parameters are fitted using the EM algorithm [42,43] which optimises the log-likelihood criterion with unknown model parameters (A,C,Q,R). Filter predictions are hidden (of filtered) state signals for a given list of observations. In our experiments, we use the pykalman library [44]. Filtering is the final common step for graph fitting and sparse CNN output.

The following section presents the description and results of the experiments.

## 3. Experimental Results

### 3.1. Preparation of Experiment

In the experimental part, we prepare the learning and testing sequence, and we learn two models based on the learning sequence: sparse CNN and graph fitting. Next, we compare models on the testing data set. During the experiments, we collected in parallel two kinds of data: (1) data from cameras (depth and RGB), and (2) the feedback information about the robot state (angles and positions) taken directly via robot API. These two kinds of signals have different time sequences of measures. The information about a robot’s state was taken every 200 ms (every data point was marked with a timestamp). Then, the state information was interpolated to the timestamps of the frames received from the cameras (also marked with the timestamps).

The training sequence included 4240 images, and it was recorded while the robot performed motion along a grid of angles. The trajectory was chosen in such a way to cover most of the working area. For learning purposes and to avoid over-fitting, we divided the training data set into two parts for learning and validation in proportion; 80%:20%. In the learning part, we added a simple data augmentation by adding a certain number of images with the salt-and-pepper noise to the data set. The test data set is an independent data set (contains 980 frames), and it was recorded after training. During the testing trajectory, the robot arm drew a sequence of squares. We used an observation sequence with 6 FPS to reduce the number of frames in all cases. The time of observations was about 700 s for training and 160 s for testing.

In experiments, we recorded two video sequences from two independent devices placed at around 1 metre from the robot arm and a distance of about 60 degrees between them, as shown in Figure 1 (marked as Left and Right camera). The test trajectory was prepared in such a way that, on a particular part of the trajectory, the robot arm was directly in front of the left camera (Figure 1). It was a difficult case to measure, but we would like to check our solution’s robustness.

We performed experiments with an i7-8700 CPU machine (3.20 GHz, 16 GB RAM) with a one-thread model and without the help of a GPU. We used the Python framework, with a machine learning part in the scikit-learn package [40] and some image processing using the OpenCV library [37]. Convolutional neural networks were developed in PyTorch [24], and using the spconv library [26], we added sparse 3D convolutional and pooling layers to the CNN PyTorch model.

### 3.2. Results

**Figure 9:**
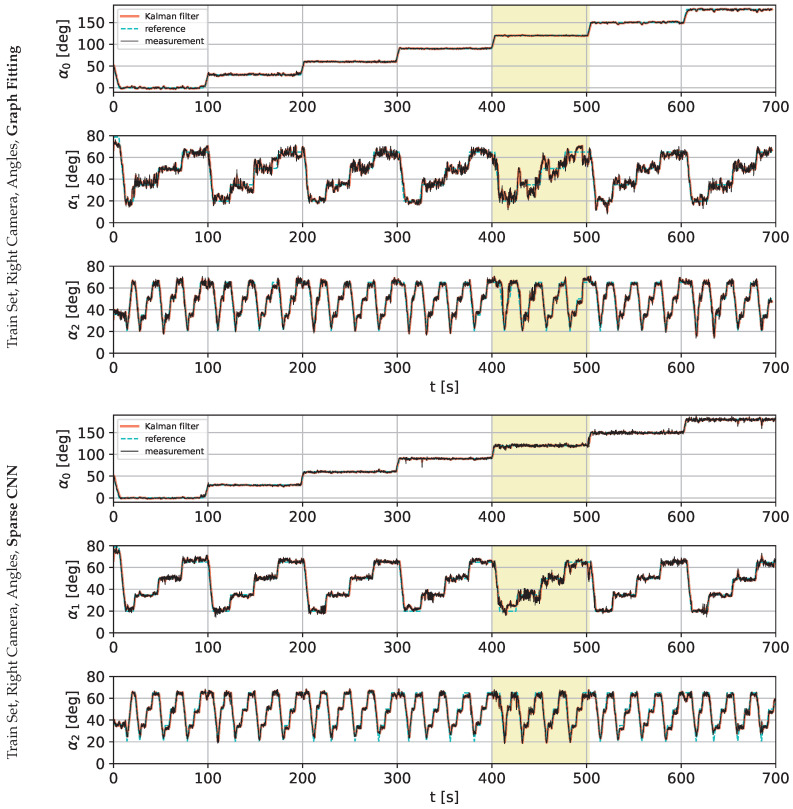
Comparison of the angle prediction accuracy for graph fitting and sparse CNN on the training data set, learned on the image from the right camera. The shaded area indicates that the robotic arm is in front of the camera, and it may cause a loss of accuracy.

**Figure 10:**
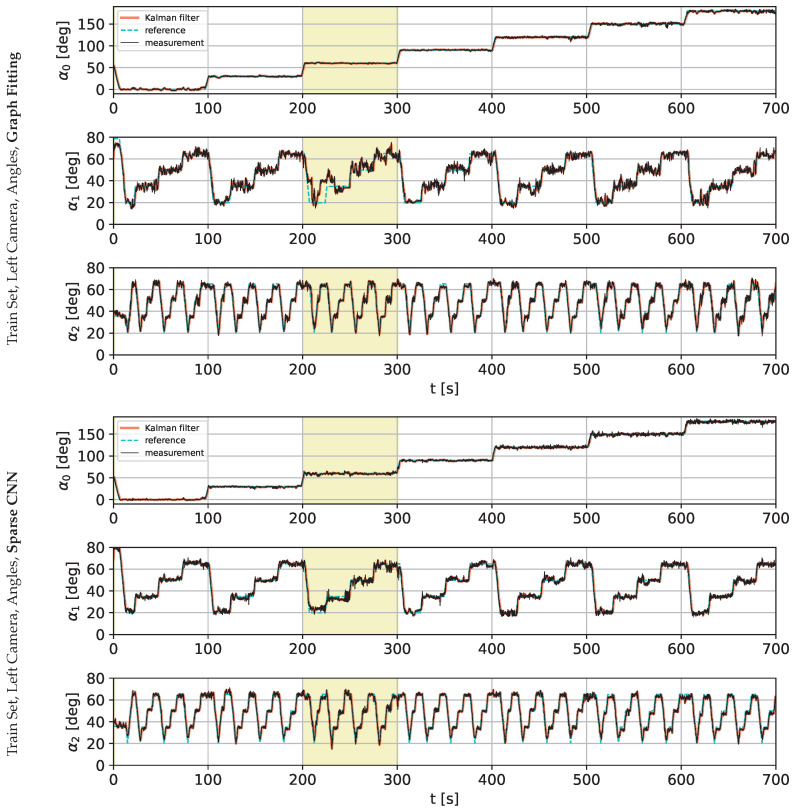
Comparison of the angle prediction accuracy for graph fitting and sparse CNN on the training data set, learned on the image from the left camera. The shaded area indicates that the robotic arm is in front of the camera.

**Figure 11:**
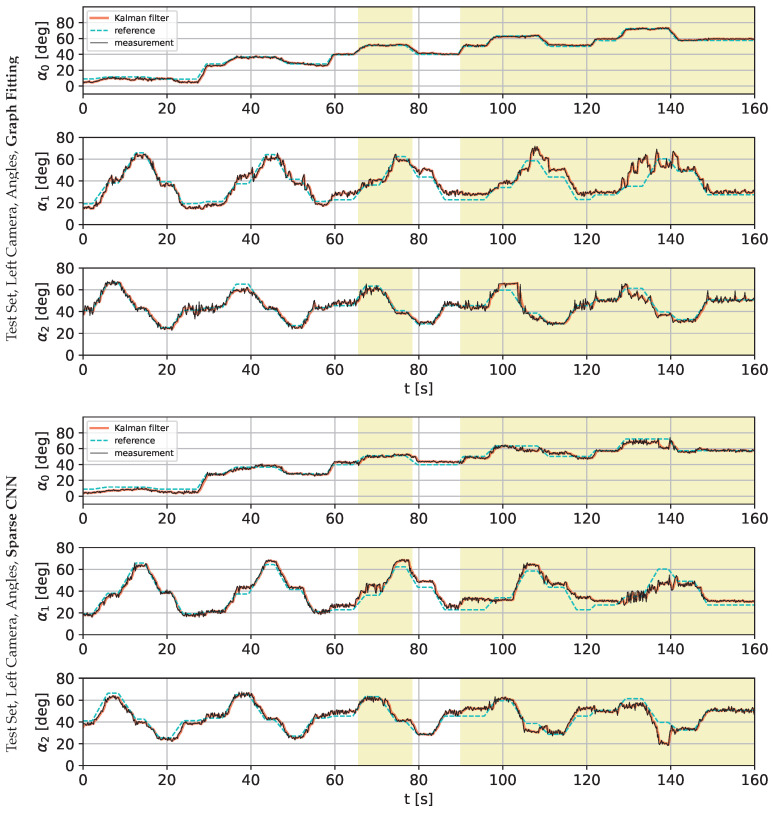
Comparison of the angle prediction accuracy for graph fitting and sparse CNN on the testing data set, learned on the image from the left camera. The shaded area indicates that the robotic arm is in front of the camera.

**Figure 12:**
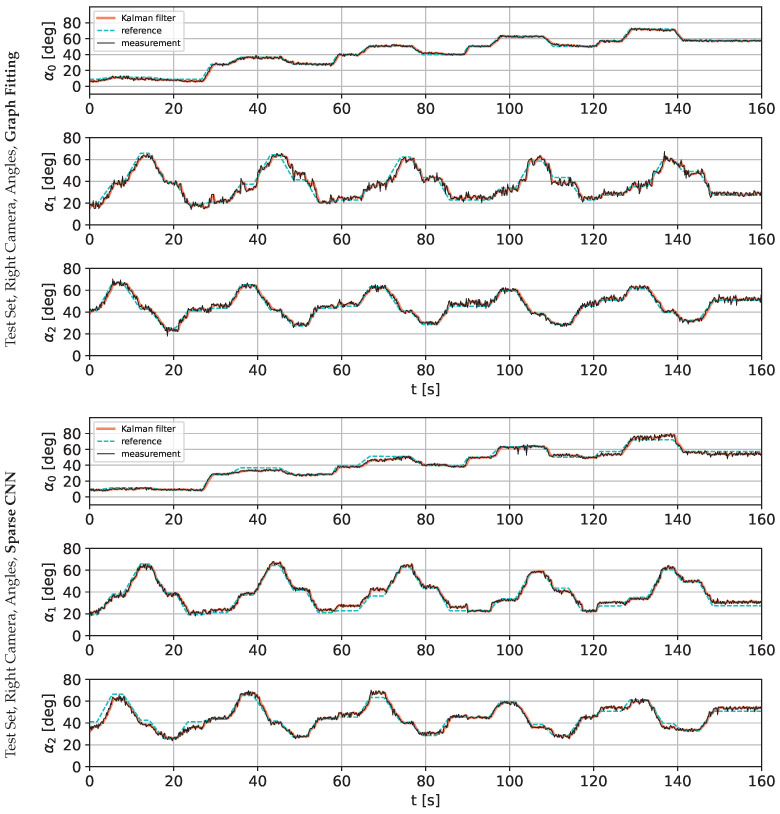
Comparison of the angle prediction accuracy for graph fitting and sparse CNN on the testing data set, learned on the image from the right camera.

**Figure 13:**
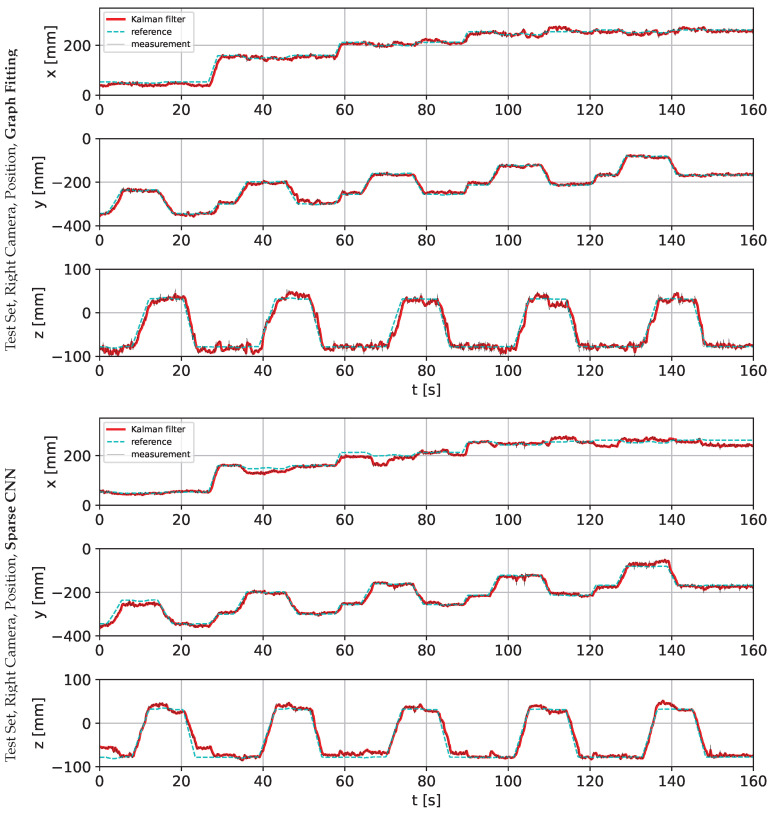
Comparison of the robot position prediction accuracy for graph fitting and sparse CNN on the training data set, learned on the image from the right camera. The shaded area indicates that the robotic arm is in front of the camera.

**Figure 14:**
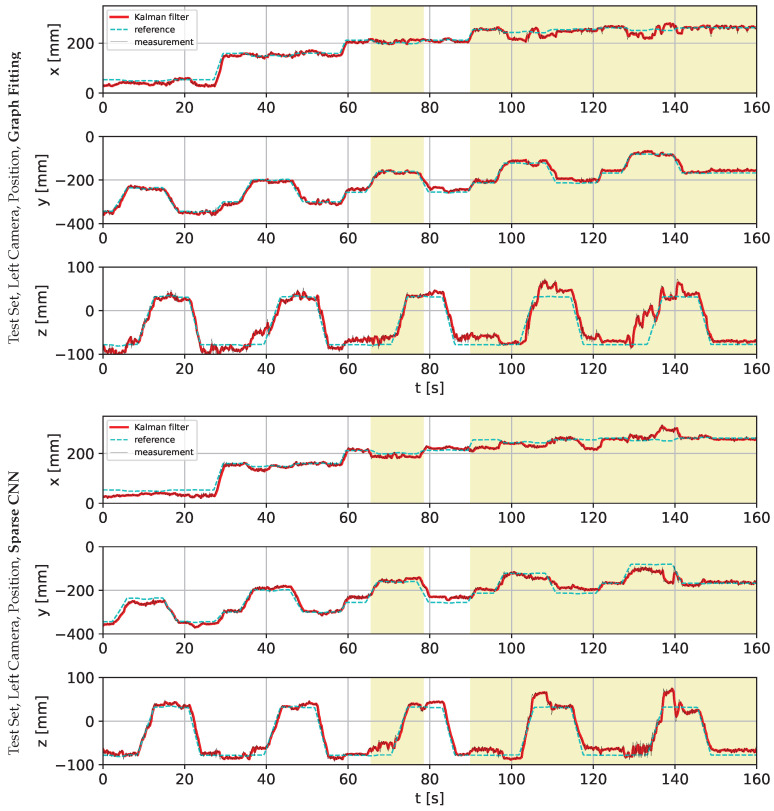
Comparison of the robot position prediction accuracy for graph fitting and sparse CNN on the testing data set, learned on the image from the left camera. The shaded area indicates that the robotic arm is in front of the camera.

**Figure 15:**
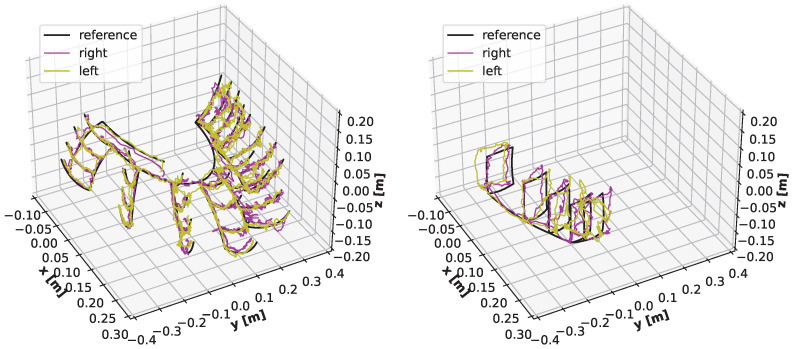
Trajectories in Cartesian space for the sparse CNN model. Black: set trajectory; colours: predictions from the left (yellow) and the right (magenta) camera.

**Figure 16:**
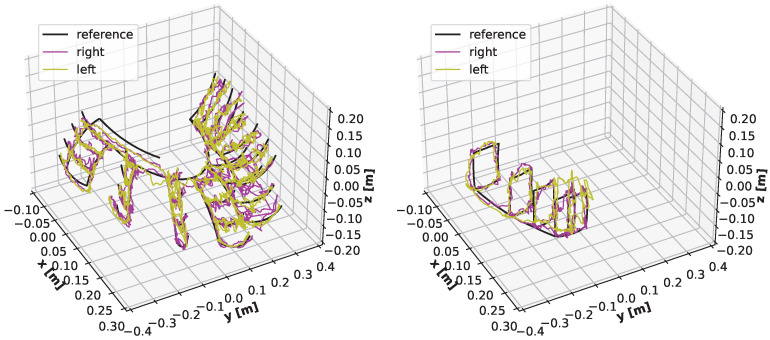
Trajectories in Cartesian space for the graph fitting model. Black: set trajectory; colours: predictions from the left (yellow) and the right (magenta) camera.

The following charts present the results and illustrate the solution’s quality for predicting the robot state (given by three angles) and the robot position. Figure 9 and Figure 10 present a detailed comparison of estimation of the robot state on learning data for the graph fitting model and the sparse CNN model. Similar comparisons of the test data are presented in Figure 11 and Figure 12. Shaded areas indicate that the robotic arm is in front of the camera; one may expect reduced accuracy in such a region. Additionally, in Figure 13 and Figure 14, we present the accuracy of the prediction of robot position. A qualitative comparison of the accuracy for the whole training trajectory is presented in Figure 15 and Figure 16. A quantitative comparison of the accuracy of the two models for robot state and positions is presented in Table 3.

As expected, the accuracy of the test set for the left camera is a bit lower than that from the right camera. The camera is in front of the robot arm in the first case. In the second case, the camera looks from the side. The mean accuracy for angle estimation is less than 3 degrees, and the accuracy of estimation of the robot position is less than 20 mm.

Table 2 and Table 4 provide detailed information about the computation time divided on subsequent operations. As we can see, preprocessing time with getting a frame from the camera takes about 12 msec. For the sparse CNN model, we obtain the speed of observation of about 50 FPS, and for the graph fitting model the speed is about 30 FPS.

## 4. Discussion

In the paper, we presented two approaches to tracking a robot pose. The first one used graph fitting, and it is an unsupervised approach. We can use it even when we do not know the exact angles. Experiments show that this approach has somewhat better accuracy than the accuracy of the sparse CNN model. The presented accuracy is about $1.8^{\circ }$–$3.3^{\circ }$ degrees for detecting angles using the graph fitting approach and $2.6^{\circ }$–$3.3^{\circ }$ (similar or slightly worse) for the convolutional network (sparse CNN).

A distance measurement accuracy for the depth sensor D435 is about 2% of the measured distance (as reported in [2]). In the scale of our experiment, this accuracy is about 1–2 cm. As declared by the producer of uArm Swift Pro, the positioning accuracy is about 0.2 mm. For both models, the accuracy of the measurement of the robot’s position is in the range of 10–20 mm. This accuracy is at the level of the accuracy of the D435 sensor (or even a bit below). The experiments show that the presented solution is a real-time solution with a speed of tracking from about 30 FPS (for a graph fitting approach) to 50 FPS (for a sparse CNN model). We should mention that accuracy and speed of computations may be too low for the feedback control purposes. In this case, the robot’s built-in angle sensors are much more accurate and faster. However, the presented approach may be acceptable for some tasks, e.g., trajectory planning or as an additional loop for safety purposes. In this paper, we show that we can observe the robot state with speed and accuracy comparable to RGB-D camera parameters. The presented approach may also be attractive for higher DOF cases when we do not have complete or accurate feedback about the robot’s state, e.g., a drone in closed areas or a robotic arm on the mobile platform. However, in such cases, direct application of sparse CNNs may be inaccurate and may need an additional object detection step.

There is some potential possibility to speed up the solution, especially for the *k*-means and the graph fitting model. There is also a clear possibility to speed up the prediction of sparse CNN models. In experiments, we used only a one-thread CPU model; using more threads or GPU can easily speed up computations. Another possibility to speed up measurements is using the event camera. The output from event cameras gives a similar representation to the sparse representation used in our approach, thus it is one possible extension of the presented research.

The graph fitting approach is unsupervised, and we can use it even when we do have not an exact measurement of angles. The restriction of this method is the computation time for more complicated (higher DOF) graph structures. The sparse CNN is a clear supervised approach, and it also has some restrictions, for example, in such tasks as human hand or body tracking or tracking drones in closed areas, while labelling data is an additional challenge. We can combine both methods for learning the targets for CNNs using graph fitting in an offline way. We also presented that the data from depth sensors in the form of the 3D point cloud can be quickly processed in a convolutional network using existing libraries. The only steps needed are transforming a point cloud to a sparse array and direct use of sparse layers.

## Figures and Tables

**Table 1:** Background removing procedure description.

Training subtractor	Collecting all grey-scaled and resized colour images from the training set. For each pixel: do *k*-means with k=2 store and identify the *k*-means result as negative pixels (background) and positive pixels (motion). Create a background mask by subtracting the positive and negative images and binarise the result to the threshold.
Removing background	Resize down the input image to the size of the positive and negative images. Select a threshold; points below the threshold are thrown away. Mark as static background pixels those whose distance from the background mask is greater than the motion mask. Resize the image to native resolution. Apply the background mask to the depth image and remove background pixels. Perform transformation depth image to the 3D point cloud.

**Table 2:** Influence of the number of points used in the *k*–means algorithm ($N_{kmeans}$) and the number of intermediate points in the graph ($N_{graph}$) on time and computation quality. (${err}_{\alpha }$ means the mean absolute error for each alpha in comparison to the real settings, ${\bar{err}}_{\alpha }$ is the mean error for three angles.)

			err*_α_* [degree]			${\bar{\mathrm{err}}}_{\alpha }$
$N_{\mathrm{kmeans}}$	$N_{\mathrm{graph}}$	Time [ms]	$\alpha_{0}$	$\alpha_{1}$	$\alpha_{2}$	[deg]
20	3	28.67	1.25	4.05	2.45	**2.58**
	4	28.32	1.24	3.99	2.35	2.53
	5	28.48	1.20	4.08	2.37	2.55
	6	28.47	1.24	4.05	2.43	2.57
40	3	31.66	1.19	3.66	2.22	2.36
	4	31.91	1.21	3.61	2.26	2.36
	5	31.80	1.20	3.72	2.17	**2.36**
	6	31.86	1.14	3.65	2.27	2.35
60	3	34.98	1.20	3.52	2.24	2.32
	4	35.12	1.15	3.69	2.22	2.35
	5	35.01	1.17	3.55	2.23	2.32
	6	34.99	1.19	3.49	2.24	**2.31**

**Table 3:** The accuracy for two models is compared: Graph fitting and sparse CNN on the training and test data sets. ($\alpha_{0}$, $\alpha_{1}$, $\alpha_{2}$, *x*, *y*, *z* are mean absolute errors for angles and positions in comparison to a real robot position; ${\mathrm{err}}_{\alpha }$ is averaged value; ${\mathrm{err}}_{\mathrm{pos}}=\sqrt{x^{2}+y^{2}+z^{2}}$ is error of position.)

			$\alpha_{0}$	$\alpha_{1}$	$\alpha_{2}$	x	y	z	${\mathrm{err}}_{\alpha }$	${\mathrm{err}}_{\mathrm{pos}}$
Camera	Model	Set	[degree]			[mm]			[deg]	[mm]
left	deep	train	1.14	1.58	1.90	5.80	6.18	6.14	1.54	10.46
		test	2.68	3.95	3.06	12.54	14.98	9.75	3.23	21.84
	graph	train	1.01	2.52	2.01	6.83	6.09	8.38	1.85	12.41
		test	1.49	4.42	2.00	9.38	9.27	12.73	2.64	18.32
right	deep	train	0.98	1.63	2.16	6.09	5.64	6.88	1.59	10.78
		test	2.06	2.25	2.23	9.77	8.48	6.85	2.18	14.64
	graph	train	0.87	2.79	2.14	6.91	5.61	8.79	1.93	12.51
		test	1.08	2.58	1.68	6.61	6.48	7.22	1.78	11.74

**Table 4:** Processing time divided into subsequent operations according to the pipeline presented in Figure 2.

Model	Task	Mean Time per Frame [ms]
Preprocessing	Getting frame from camera	7.07
(common part)	Background subtraction	1.20
	Point cloud from depth image	4.46
	Subtotal time:	**12.73**
Graph	*k*–means	8.55
	Graph fitting	10.47
	Transformation + Regression	≪1.00
	Total time:	**31.76**
Sparse CNN	Prediction	7.03
	Total time:	**21.94**

## Data Availability

The data set was recorded during an experiment in our laboratory. Video sequences and other data used in experiments are available at https://edysk.zut.edu.pl/index.php/s/yLBiJPH6WGAG2t6 (accessed on 24 August 2022). For results replication, please follow https://github.com/jrod12/py_realsense_sensors (accessed on 24 August 2022) and readme.md file.

## Abbreviations

The following abbreviations are used in this manuscript:

MDPI	Multidisciplinary Digital Publishing Institute
DOAJ	Directory of open access journals
CNN	Convolutional Neural Network
Sparse CNN	CNN with sparse convolutional and pooling layers and with sparse inputs
Graph Fitting	Model fitting nodes of graph to the point cloud
FPS	Frames per Second
MLP	MultiLayer Perceptron
SGD	Stochastic Gradient Descent
RGB-D	RGB-Depth, combination of RGB (standard) image and depth image
RBF	Radial Basis Function
MAE	Mean Absolute Error
MSE	Mean Squared Error
GPU	Graphics Processing Unit
CPU	Common x86-64 processor
DOF	Degrees of Freedom
